# Mom—It Helps When You're Right Here! Attenuation of Neural Stress Markers in Anxious Youths Whose Caregivers Are Present during fMRI

**DOI:** 10.1371/journal.pone.0050680

**Published:** 2012-12-07

**Authors:** Olivia L. Conner, Greg J. Siegle, Ashley M. McFarland, Jennifer S. Silk, Cecile D. Ladouceur, Ronald E. Dahl, James A. Coan, Neal D. Ryan

**Affiliations:** 1 University of Pittsburgh Medical Center, Pittsburgh, Pennsylvania, United States of America; 2 University of Pittsburgh School of Medicine, Pittsburgh, Pennsylvania, United States of America; 3 University of California, Berkeley, California, United States of America; 4 University of Virginia, Charlottesville, Virginia, United States of America; Federal University of Rio de Janeiro, Brazil

## Abstract

Close proximity to an attachment figure, such as a caregiver, has been shown to attenuate threat-related activity in limbic regions such as the hypothalamus in healthy individuals. We hypothesized that such features might be similarly attenuated by proximity during a potentially stressful situation in a clinically anxious population of youths. Confirmation of this hypothesis could support the role of attachment figures in the management of anxiety among children and adolescents. Three groups were analyzed: anxious children and adolescents who requested that their caregiver accompany them in the scanner room, anxious children and adolescents without their caregiver in the scanner room and healthy controls (each of N = 10). The groups were matched for age and, among the two anxious groups, for diagnosis (mean age 9.5). The children and adolescents were exposed to physical threat words during an fMRI assessment. Results indicate that activity in the hypothalamus, ventromedial, and ventrolateral prefrontal cortex were significantly reduced in anxious children and adolescents who requested that their caregiver accompany them in the scanner room compared to those without their caregiver in the scanner room. Mean activity in these regions in anxious children and adolescents with their caregiver in the scanner room was comparable to that of healthy controls. These data suggest links between social contact and neural mechanisms of emotional reactivity; specifically, presence of caregivers moderates the increase in anxiety seen with stressful stimuli. Capitalizing on the ability of anxious youths to manifest low levels of anxiety-like information processing in the presence of a caregiver could help in modeling adaptive function in behavioral treatments.

## Introduction

This study examined attenuation of neural features of reactivity to threat in anxious youths by social proximity to an attachment figure. Traditionally, it is believed that high levels of reactivity to threat is inherent, and indeed, diagnostic for anxiety disorders [1]. Given the likelihood that social proximity can attenuate the core features of anxiety, this has crucial implications for how to investigate and understand the essential elements of anxiety.

There are strong reasons to believe that social proximity could attenuate youth anxiety. For example, socially supportive relationships have been linked with decreased cardiovascular stress-responses as well as decreased resting stress hormone levels [2]. By way of explanation, proximity seeking behavior as early as infancy has been considered an inborn affect-regulation device responsible for the alleviation of distress and the protection from physical and psychological threat [3]. Social contact has been hypothesized to more generally support enhanced health and well-being and to ease distress [3], possibly by moderating harmful health effects of psychosocial stress [4]. In lay terms, social support could help by giving one “a shoulder to cry on, a hand to hold, an ear to listen to you, someone to cradle you and tell you it will be okay” [2].

The extent to which such effects attenuate mechanisms of clinically relevant anxiety in youths during an fMRI scan is unclear. Anxiety is associated with selective processing of emotional information; particularly, increased processing of threat related stimuli [5]. Initial data, upon which this study was based, suggest that neural mechanisms of attention to threat cues may be reduced by social proximity. Coan et al. [6] showed that compared to a non-hand-holding condition, neural response to threat during fMRI assessment was attenuated in a network associated with emotion regulation in married women allowed to hold the hand of either their spouse or a stranger (ventral anterior cingulate, supramarginal gyrus) and specific to the spouse hand-holding condition in regulatory, perceptual, and limbic regions (dorsolateral prefrontal cortex, caudate and superior colliculus). Attenuation in regions associated with low-level emotional and perceptual information processing was sensitive to marital quality (right anterior insula, superior frontal gyrus and hypothalamus). These results suggest that hand-holding is associated with reduced neural reactivity to threat responses and that these reductions are dependent on social knowledge. Importantly, as activity in virtually no regions increased with hand-holding there was no sense in which social proximity increased regulation. Rather, social proximity can be construed as reducing natural tendencies towards reactivity [7].

Thus, the current study focused on three *a priori* regions associated with both perception and processing of threat and anxiety including the hypothalamus, the ventrolateral prefrontal cortex (VLPFC) and the ventromedial prefrontal cortex (VMPFC). The hypothalamus was chosen as an *a priori* region based on a study by Coan et al. [6], which found that the hypothalamus, among other areas, was attenuated when compared to a non-hand-holding condition in response to threat. The other two regions were considered regions to specifically follow up on if they emerged empirically upon voxelwise analysis, based on their proximity to other areas identified by Coan et al. [6] as well as their association with processing of threat and anxiety. The hypothalamus functions as the control center for most of the body's hormonal systems and as a key component of the hypothalamic-pituitary-adrenal (HPA) axis. In reacting to stress, hormones released by the hypothalamus cause a chain of events that eventually end in the production and circulation of cortisol throughout the body and brain [8]. The VLPFC has been implicated in vigilance for threat [9]. Youths with generalized anxiety disorder display greater activation of the right VLPFC when compared with healthy peers [10]. The VMPFC is also thought to mediate HPA axis disturbances related to chronic stress and cortisol secretion [11]. The VLPFC and VMPFC have also been implicated in emotion regulation [11].

To examine the role of social contact in clinically relevant anxiety, in this study, we examined clinically anxious children and adolescents during their identification of the emotionality of age-appropriate physical threat words during fMRI. Some of the children and adolescents requested that their parent be in the room, thus yielding social proximity during the scan, while others did not. We analyzed effects of social proximity on brain activity of clinically anxious compared to healthy children and adolescents during this stressful situation (presentation of threat stimuli). We hypothesized that social proximity during a potentially stressful situation would attenuate activity in the hypothalamus in clinically anxious youths. As noted previously, the VMPFC and VLPFC have been associated with both reactivity and regulation. Following [7], we will interpret increases in activity in these areas with social contact as reflecting increased broad neural response to threat, likely encompassing aspects of both reactivity and regulation, and decreases in activity in these areas with social contact as reflecting decreased neural response to threat consistent with a lack of reactivity and associated need for regulation. In either case, if activity is increased, we can suggest that the presence of a caregiver facilitates neural response to threat. If it is decreased, we can say that, as proposed by Beckes and Coan [7], caregivers decrease the need for neural response to threat involving emotional reactions and their regulation.

## Methods

### Participants

In a larger study of anxiety among children and adolescents (henceforth, youths) (ClinicalTrials.gov, identifiers: NCT00774150), 10 youths with an anxiety disorder (GAD, SAD, Separation anxiety) requested their caregiver in the scanner room with them to date (5 male, 5 female, mean age 9.5, S.D. 0.71). These caregivers were consistently located in the corner of the scanner room. Youth and caregiver were not making physical contact during the scan. We randomly matched these participants on age, gender and exact diagnosis within one year to 10 youths with anxiety disorders who did not request their caregiver in the scanner room with them (from a larger sample of about 80 youths) and to 10 healthy controls matched on age (from a larger sample of about 30 youths) (3 male, 7 female). The caregivers of the youth that did not request their caregiver in the scanner room with them were in the in the waiting room during the scan. The participants without anxiety were matched on age but not gender because there were not enough male participants within the relevant age ranges. Of the 10 youths that requested a caregiver in the scanner room, 8 were accompanied by a mother, one by a father and one by a grandmother.

Participants were recruited from the community through radio, television, and newspaper advertisements, referrals from pediatricians and school counselors, and other University mental health clinics and research studies. Anxious youths were required to meet DSM-IV criteria for current generalized anxiety disorder (GAD), separation anxiety disorder (SAD), social phobia (SP) or a combination of these disorders (see Table 1). Exclusion criteria for all participants included an IQ below 70 as assessed by the Wechsler Abbreviated Scale of Intelligence [12], use of ongoing treatment with psychoactive medications, acute suicidality or risk for harm to self or others, and presence of metal braces or other metal objects in their body. Specific exclusion criteria for healthy comparison youths included any current or lifetime DSM-IV diagnosis and having a parent with a current or lifetime DSM-IV diagnosis of anxiety or mood disorders. Specific exclusion criteria for anxious participants included a current primary diagnosis of major depressive disorder (MDD), a current diagnosis of obsessive-compulsive disorder (OCD), post-traumatic stress disorder (PTSD), conduct disorder, substance abuse or dependence, and ADHD combined type or predominantly hyperactive-impulsive type. Anxious youths were also excluded if they had a lifetime diagnosis of autism or Asperger syndrome, bipolar disorder, psychotic depression, schizophrenia, or schizoaffective disorder.

**Table 1:** Frequencies for diagnoses of anxious youths in each group (matched by design).

	GAD	SAD	SP	GAD/SAD	GAD/SP
**With Parent**	5	2	1	1	1
**Without Parent**	5	2	1	1	1

### Procedure

This study was approved by the University of Pittsburgh Institutional Review Board. Youths and their parents gave written assent and informed consent, respectively. Participants completed a large battery of questionnaires and ratings. Two measures relevant to this project included the Screen for Child Anxiety Related Emotional Disorders (SCARED) and the Pediatric Anxiety Rating Scale (PARS). Participants and their parents completed the Screen for Child Anxiety Related Emotional Disorders [13] and clinicians completed Pediatric Anxiety Rating Scale [14]. The Screen for Child Anxiety Related Emotional Disorders consists of 41 questions and functions as a youth and parent self-report instrument to screen youths with anxiety disorders. The Pediatric Anxiety Rating Scale consists of a 0–7 scale and functions as a clinician-report measure of the severity of anxiety symptoms in youths.

### Apparatus

Thirty-two 3.2 mm slices were acquired parallel to the AC-PC line using a reverse direction EPI pulse sequence to minimize susceptibility artifacts in the amygdala and orbitofrontal regions (3T Siemens Trio, T2*-weighted images depicting BOLD contrast; TR = 1670 ms, TE = 25 ms, FOV = 24 cm, flip = 80), yielding 7 whole-brain images per 11.69 second trial. Stimuli were displayed in dark grey on a light grey background via a back-projection screen. Responses were recorded using a Psychology Software ToolsTM glove. Mappings of glove buttons to responses were counterbalanced across participants and displayed throughout the tasks (e.g., “+N−” representing “Positive” on the index finger, “Neutral” on the middle finger and “Negative” on the ring finger).

### Word Valence Identification (VID) Task

During fMRI scanning, participants completed a word VID [15]. Participants were instructed to identify the emotional valence of physical threat, social threat, positive and neutral words by pressing a corresponding button as quickly and accurately as possible. Using the three-button glove described above, participants pressed the button that corresponded with their rating of the word as positive, negative, or neutral. The physical threat (i.e., ghost), social threat (i.e., embarrassed), positive (i.e., laughing) and neutral (i.e., grape) words used in the word VID task were chosen from a corpus of emotional words normed for use with youths [16]. Fifteen words from each valence category were selected and balanced for word length and frequency. Each trial included a 1000 ms cue (a row of Xs flanked by prongs), presentation of the word for 1500 ms, and a mask (another row of Xs) for the 9190 ms inter-trial interval.

### Data Selection and Cleaning

Functional MRI data was prepared using time-slice correction (AFNI TimeSlice), motion correction (AFNI 3dVolReg [17]), detrending (NISCorrect), temporal smoothing with a five-point middle-peaked filter (NISconv), voxelwise conversion to percent-change from the data-set's median, cross-registration (AIR 32-parameter non-linear warp to MNI brain [18]), spatial smoothing (6 mm FWHM, NIS gsmooth), and voxelwize standardization of “reactivity” to threat stimuli, operationalized as activity in the 3–4^th^ scans following physical threat stimuli minus a prestimulus baseline, to normalized scores with respect to control participants (i.e., X-mean(X_controls_)/std(X_controls_); custom Matlab code). Responses to social threat, positive and neutral words were not examined for this study. Following this standard preprocessing, we analyzed group differences in the standardized reactivity contrast using voxelwise t-tests (AFNI 3dTtest) for anxious youths without a caregiver compared to 1) anxious youths with a caregiver and 2) controls. To preserve voxelwise *p*<.01, the conjunction of these two t-tests, each tested at *p*<.09 was assessed as a measure of unique features of social proximity, yielding multiplicative probably of co-activation .09*.09 = .0081. Significant voxelwise tests within a gray matter mask were subjected to an empirically determined contiguity threshold based on the spatial autocorrelation of the with-vs-without parent statistical map (55 voxels) yielding a corrected *p*<0.05. Given cytoarchitectonic and functional differentiation within prefrontal regions of interest, significant prefrontal clusters were separated after contiguity thresholding into a ventrolateral region (Talairach x<−10.5) and a ventromedial region (Talairach −10.5<x<10.5). Hypotheses about the hypothalamus were tested at *p*<0.05 uncorrected, within the AFNI Talairach-derived hypothalamus mask, as it is such a small region.

## Results

### Symptom contrasts

SCARED scores did not differ between the anxious groups for youth-ratings, *p*>0.4 as well as parent ratings, *p*>0.5. Also, PARS scores did not differ, *p*>0.9 (see Table 2).

**Table 2:** Symptom contrast of anxiety severity scores SCARED (youth and parent rating) and PARS between anxious groups.

	SCARED: youth-rating	SCARED: parent-rating	PARS
**With Parent**	39.00	4.10	19.89
**Without Parent**	34.42	4.00	20.10
**Significance (** ***p*** **)**	0.416	0.573	0.930
**t**	0.834	0.578	−0.090
**df**	17	14	17

### fMRI data

**Figure 1:**
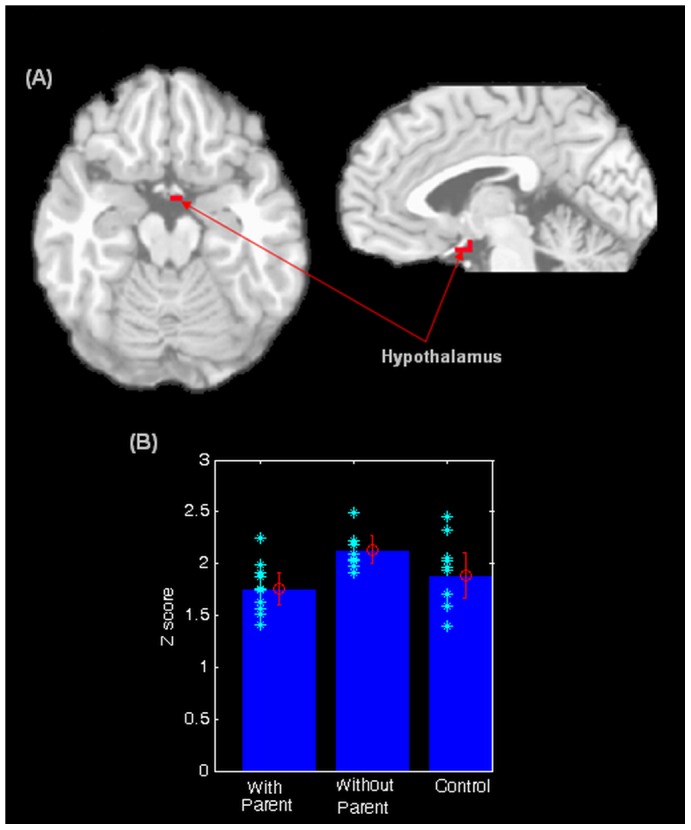
*a*. Significant difference observed in hypothalamus among anxious with caregiver group, anxious without caregiver group and controls. *b*. Z-scores of hypothalamic activity among anxious with caregiver group, anxious without caregiver group and controls.

**Figure 2:**
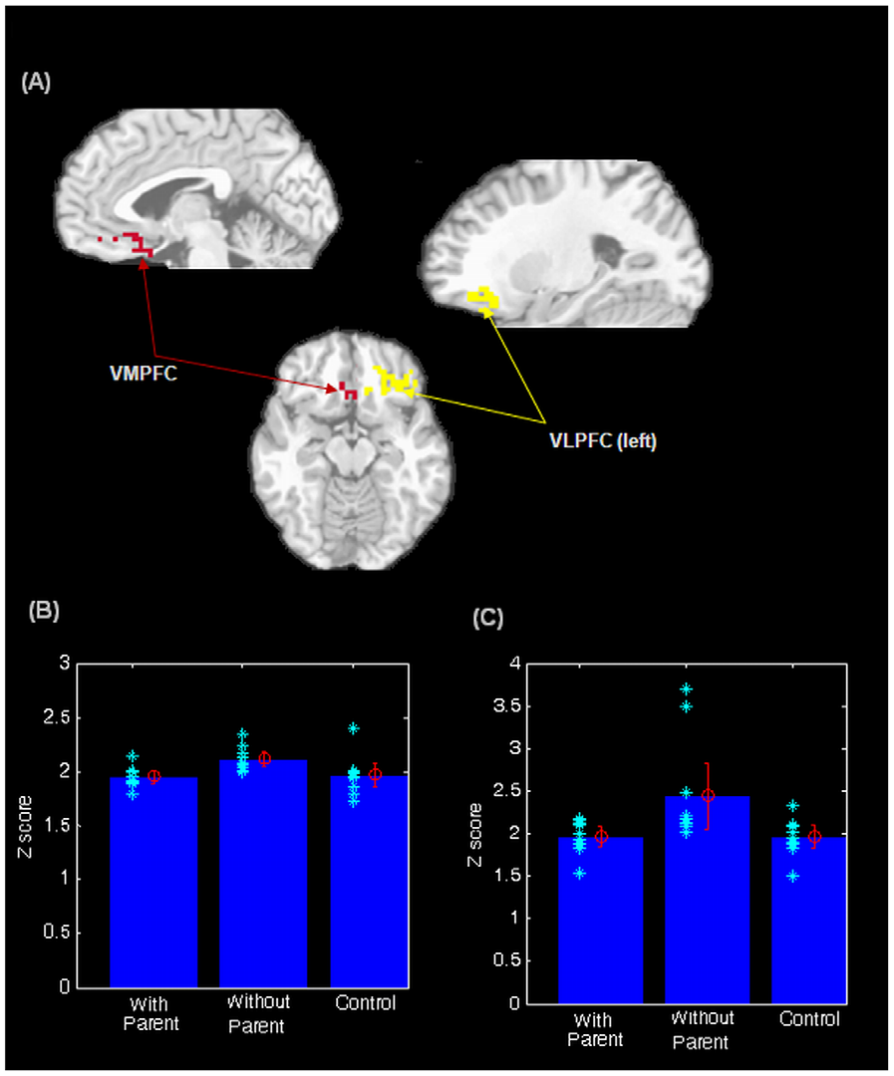
*a*. Significant differences observed in ventral prefrontal regions (VMPFC and VLPFC-left) among anxious with caregiver group, anxious without caregiver group and controls. *b*. Z-scores of VMPFC activity among anxious with caregiver group, anxious without caregiver group and controls. *c*. Z-scores of VLPFC-left activity among anxious with caregiver group, anxious without caregiver group and controls.

Significant effects of caregiver presence among anxious youths were found in the hypothalamus, VLPFC (left) and VMPFC (see Table 3 and Figures 1a and 2a). Activity in all three regions was significantly reduced in anxious youths with caregivers present compared to anxious youths with caregivers absent. Mean activity in anxious youths with caregivers present was comparable to that of controls (see Figures 1b, 2b, 2c).

**Table 3:** Regions associated with reduced activity in association with social proximity.

Region	Location of centroid voxel	Brodmann's areas	x	y	z	Cluster extent (mm^3^)
Left Ventrolateral PFC	Inferior Frontal Gyrus	47	−31	31	−6	3924
Ventromedial PFC	Anterior Cingulate	25	−1	20	−9	1080
Left Hypothalamus	Hypothalamus	–	−2	−1	−12	282

*Note:* Coordinates for each cluster's center-of-mass are presented in Talairach space.

### Sensitivity analysis

To examine the extent to which significant contrasts were a function of the specific youths chosen from the larger sample and matched with the anxious youths with caregivers present, we examined substitutions with all other potential matches from the other two groups from the larger sample of 82 anxious youths who did not request parents and 31 controls. This procedure was used to preserve strict matching on age, gender, and diagnosis along with equal n's, which was crucial given the small sample sizes. There were ten possible matches for members in the original data set (anxious without caregiver present and controls) yielding nineteen possible substitution sets for examined participants; all were analyzed. VLPFC (left) ROI voxels were significant in a maximum of 17/19 substitution sets, M(SD) = 4.3(4.7) sets. Voxels in the VMPFC mask were significant in a maximum of 17/19 substitution sets, M(SD) = 8.1(5.7) sets. Hypothalamus ROI voxels were significant in a maximum of 16/19 substitution sets, M(SD) = 12.8(3.1) sets. These data suggest robustness across the sampled population-subset in partial volumes of the examined structures, with slightly different extents.

## Discussion

As hypothesized, social proximity to a caregiver during a potentially stressful situation attenuated activity in the hypothalamus, VMPFC, and VLPFC (left) in clinically anxious youths. These results are consistent with earlier research [6], suggesting that hypothalamus and ventral prefrontal activity during a stressful situation are decreased in the presence of a loved one. Also, Coan et al. [6] found that hypothalamic attenuation was dependent upon the quality of the relationship with the hand holder. A similar process could be occurring here, and could explain the results of the sensitivity analysis in the hypothalamus.

As activity decreased for all three structures it is unlikely that these results reflected regulatory influences of the hypothalamus by the ventral prefrontal cortex but rather, as suggested by Beckes and Coan's Social Baseline Theory [7], these data could suggest a “return” to a baseline state of low reactivity. To the extent that this baseline represents a positive motivational state, as opposed to an anxious threatened state, these data are consistent with a positivity offset [19] or the general expectation of positive outcomes for unknown events, in the absence of a specifically stressful stimulus.

A baseline state would include proximity to trusted members of one's social network, and failing this proximity, negative affect should arise in proportion to perceived situational demands. Social proximity would provide the individual with a sense of the environment to which he or she is already adapted, and therefore allow the individual to be less vigilant to potential threats as perceived contextual demands decrease. Less vigilance would translate into less emotional regulation as well as less self-regulatory inhibition. In a sense, social regulation effects do not down-regulate affect so much as dyadically remove the need for the generation of negative affect.

Here, the presentation of threat stimuli can be seen as a threatening situation. Youths with an anxiety disorder would, in theory, be using all their regulatory mechanisms to cope with their anxiety and maintain their baseline state. When these individuals are subjected to a stress inducing event, such as viewing threatening stimuli, their resources may already be partially depleted from regulating their anxiety, and further maintenance of their baseline state will become difficult. Social proximity, however, functions as a cue, informing these individuals that more resources are available and fewer problems need to be solved. Therefore, there would be a decrease in the perceived context demand, a decrease in the use of regulatory mechanisms and a return to the baseline state. So, instead of viewing the significant differences seen in the brain activity of the youths with an anxiety disorder that requested a caregiver in the scanner room as decreases in neural activity, it could be interpreted as a return to a baseline state. Whereas, the youths with an anxiety disorder that did not request a caregiver in the scanner room have increased activity from baseline due to their lack of social proximity. fMRI scans can be considered stressful in and of themselves due to the lack of social interaction when confronting a novel, noisy and potentially threatening environment. That said, such effects are tonic and thus not likely to entirely account for the alterations in stimulus related reactivity observed in this experiment.

These data suggest changes in emotional processing in the presence of a caregiver; caregivers may act as emotion regulators. Thus, considering their role in facilitating emotion regulation could be useful in structuring treatments for anxious youths that aim to increase emotion regulatory function, e.g., by initially involving and gradually removing parental support. Barrett et al. [20] found that children with an anxiety disorder responded significantly better to cognitive-behavioral therapy plus family anxiety management training than cognitive-behavioral therapy alone based on seven clinical evaluation scales completed at posttreatment and at 6- and 12-month follow-ups.

It is important to note that the current findings may not generalize to adult populations. Further, no controls (youths without anxiety) naturalistically requested to be accompanied by a caregiver in the scanner room; it would be interesting to determine if attenuation of similar neural systems would be seen in controls with proximity to a caregiver. Another limitation is our small sample sizes, which is due to the fact that these data are from a larger study examining anxiety among youths.

Additionally, there was a non-random assignment of participants to groups (with versus without caregivers). It could be fundamental differences between the anxious youths that requested a caregiver in the scanner room and the anxious youths that did not request a caregiver in the scanner room rather than state-related potentiation. We did not identify such a variable; there were no significant differences in the severity of anxiety symptoms between these groups.

These limitations notwithstanding, our results bring to light the potential importance of social proximity in distress alleviation. Specifically, the lowest-level neural features of threat reactivity associated with anxiety in youths may be contextual, manifesting primarily in situations in which anxious youths are alone and unsupported. Helping anxious youths to feel more supported and less alone could be important to normalizing brain function in this population.
