# Correction: Mom—It Helps When You're Right Here! Attenuation of Neural Stress Markers in Anxious Youths Whose Caregivers Are Present during fMRI

**DOI:** 10.1371/annotation/f5ea136c-b1d5-4528-866e-f4f1a055ebaf

**Published:** 2013-10-17

**Authors:** Olivia L. Conner, Greg J. Siegle, Ashley M. McFarland, Jennifer S. Silk, Cecile D. Ladouceur, Ronald E. Dahl, James A. Coan, Neal D. Ryan

The second author, Greg Siegle, should be noted as a Corresponding Author for the article. The second author's email address is: gsiegle@pitt.edu

